# COVID-19 Mortality in a Pediatric Patient with Hemoglobin SC Disease and Alpha-Thalassemia Trait

**DOI:** 10.1155/2021/6617362

**Published:** 2021-04-27

**Authors:** Joshua E. Motelow, Stacie Kahn, Patrick T. Wilson

**Affiliations:** Department of Pediatrics, Division of Critical Care and Hospital Medicine, Columbia University Irving Medical Center, New York-Presbyterian Morgan Stanley Children's Hospital of New York, New York, NY, USA

## Abstract

As the pandemic continues to evolve, more cases of COVID-19 in pediatric patients are being detected. A 12-year-old boy with HbSC disease alpha-thalassemia trait presented to a pediatric emergency room with fever and weakness. His vital signs were notable for fever, tachypnea, and tachycardia. His physical exam was concerning for increased work of breathing. He tested positive for severe acute respiratory syndrome coronavirus 2 (SARS-CoV-2) by PCR although his hemoglobin level remained near his baseline. His chest radiograph showed a retrocardiac opacity concerning for evolving acute chest syndrome. He decompensated quickly requiring invasive mechanical ventilation and exchange transfusion. He received hydroxychloroquine, broad-spectrum antibiotics, and enoxaparin for DVT prophylaxis. Despite showing clinical signs of improvement, he became acutely hypoxemic and suffered a cardiac arrest. We believe this to be an unusual case of a pediatric patient with HbSC disease and COVID-19. We outline clearly the course of illness and treatments trialed, which can prove beneficial to providers facing similar challenges as this virus continues to strike areas around the world. Although children have significantly better outcomes than adults, providers must remain vigilant while treating any patient with a hemoglobinopathy in the setting of severe COVID-19.

## 1. Introduction

Children infected with the novel severe acute respiratory syndrome coronavirus 2 (SARS-CoV-2) generally fare better than adults but may suffer critical illness [1, 2]. Of those requiring admission to the pediatric intensive care unit (PICU), more than 80% carry a comorbidity including hematologic comorbidities although hematologic conditions may not be an independent risk factor of severe COVID-19 [1, 2]. Among pediatric patients, respiratory failure requiring invasive mechanical ventilation secondary to COVID-19 is rare, and a large study found a case fatality rate of 0.2% [1]. Here, we present the case of a pediatric patient with hemoglobin SC disease and alpha-thalassemia trait who tested positive for SARS-CoV-2 and died from refractory hypoxemia. The concurrence of a hemoglobinopathy and severe COVID-19 may warrant heightened clinical suspicion for unusual outcomes. We outline the course of illness and treatments trialed with the hope of improving the ability of the medical community to care for these patients.

## 2. Case Presentation

A 12-year-old male with HbSC disease alpha-thalassemia trait, with mild sickle-related complications including no history of splenectomy, presented to a tertiary hospital in the spring of 2020 with acute respiratory failure in the setting of a positive PCR for severe acute respiratory syndrome coronavirus 2 (SARS-CoV-2). The patient was evaluated in the emergency department (ED) one day prior to hospital admission for nausea, subjective fever, and left lower quadrant tenderness. However, he was afebrile, well appearing, and had a hemoglobin level near his baseline (10.6 g/dL). Based on this, he did not meet hospital testing criteria for SARS-CoV-2 at that time so he was discharged home. The following day, he developed fever, weakness, and pallor at which time he was referred back to the ED. He was noted to be febrile (39.2°C), tachycardic (116 beats/minute), and tachypneic (36 breaths/minute) with an oxygen saturation of 97%. His physical exam was notable for labored breathing. The patient tested positive for SARS-CoV-2 by nasopharyngeal PCR testing. The initial chest radiograph was notable for a retrocardiac opacity, and he received ceftriaxone and doxycycline (azithromycin was on shortage) for empiric management of acute chest syndrome (ACS). The patient required rapid escalation of respiratory support with noninvasive bilevel positive airway pressure before transfer to the PICU.

On arrival to the PICU, the patient was in respiratory extremis and emergently intubated by anesthesia using COVID-19 airway precautions: rapid sequence intubation, negative pressure room, N-95 mask, gown, gloves, eye protection, bouffant, and video laryngoscopy. Despite lack of data, hydroxychloroquine was initiated (600 mg enteral every 12 hours) following confirmation of a normal QTc, and he was started on deep vein thrombosis prophylaxis with enoxaparin (30 mg subcutaneous every 12 hours).

The patient met criteria for severe acute chest syndrome [3, 4] with a new pulmonary infiltrate on chest radiograph, fever, and respiratory failure. Given severity of illness and baseline Hb (10.7 g/dL), exchange transfusion was indicated in order to rapidly reduce the HbS% [3] so an internal jugular vascath central line was placed. On hospital day 2, the patient was exchanged with ten units of sickle-negative, C, E, and K antigen crossmatched compatible packed red blood. The pretransfusion HbS and HbC percentages were 45% and 48%, and posttransfusion percentages were both 7%. Immediately following the exchange transfusion, he became hypotensive with warm extremities requiring fluid resuscitation, epinephrine (max of 0.1 *μ*g/kg/min) and norepinephrine (max of 0.03 *μ*g/kg/min). An acute transfusion reaction was ruled out by the blood bank team. Antibiotics were subsequently broadened to vancomycin and piperacillin-tazobactam (in addition to the doxycycline) until blood cultures were negative at 48 hours. Both vasoactive medications subsequently were weaned off the following day. Following the exchange transfusion, there was an acute increase in the patient's creatinine from 0.87 mg/dL to 1.60 mg/dL meeting criteria for acute renal failure in a patient with sickle cell disease [3]. Renal function was monitored daily, nephrotoxic medications were avoided (including remdesivir), and nephrology was consulted [3].

**Figure 1:**
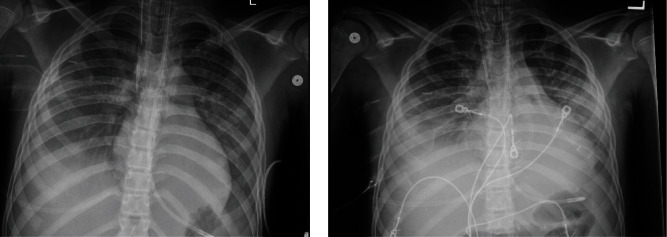
Chest radiograph one day prior (a) and at day of arrest (b).

The patient met criteria for mild pediatric acute respiratory distress syndrome (pARDS) with a positive SARS-CoV-2 test, bilateral infiltrates consistent with acute pulmonary disease, and a PaO_2_ to FiO_2_ (*P*/*F*) ratio of 287 [5, 6]. Open lung (high peak end expiratory pressure) and lung protective ventilatory strategies (small tidal volumes with peak pressures under 30 cm of water) were utilized per PICU standards [5]. On the morning of his arrest (PICU day 4), the patient's secretions were noted to be thick and copious with a chest radiograph showing worsening bilateral infiltrates (Figure 1) despite a stable *P*/*F* ratio of 253.

Routine labs obtained the morning of the arrest are shown in Table 1. The patient's hemoglobin had dropped to 8.0 g/dL from 9.3 g/dL the day prior, and his white blood cell count was stable at 23.5 × 10^3^/*μ*L without bandemia. His platelet count was 126 × 10^3^/*μ*L which was improved from the day prior. His D-dimer was 18.2 *μ*g/mL, consistent with the coagulopathy of COVID-19 but was downtrending [7]. His fibrinogen was steadily increasing from admission and was 458 mg/dL, which may have indicated worsening inflammation. Liver function tests were notable only for a low albumin (2.9 g/dL) and mildly elevated aspartate aminotransferase (93 U/L). Basic metabolic panel showed a lower creatinine of 1.33 mg/dL suggesting improving acute kidney injury.

In the early afternoon he became hypoxemic in the setting of an acute drop in tidal volumes delivered by the ventilator. Despite manual ventilation with evidence of good chest rise and 100% oxygen administration, the patient's oxygen saturations remained in the 70% range. Repeated inline suctioning and increased ventilator settings did not resolve the hypoxemia, and the patient became increasingly bradycardic; chest compressions were initiated once less than 60 beats per minute. Per local CPR protocol for COVID-19 patients, the patient remained on the ventilator while receiving compressions to avoid aerosolization of respiratory secretions. Despite chest compressions, bilateral needle thoracostomy (no rush of air noted), and multiple doses of epinephrine administration, the patient did not regain pulses. The family requested no autopsy. During the early stages of the pandemic, limited invasive postmortem investigations were done. No postmortem imaging was performed.

## 3. Discussion

Early case series of adult patients with sickle cell disease and COVID-19 report mild disease, and centers with significant sickle cell populations report minimal severe pediatric presentations supporting the hypothesis that sickle cell disease is not generally considered a risk factor for severe COVID-19 [8–11]. Supporting these case reports, a larger French study found a lower rate of intensive care unit admission for teenagers and young adults with sickle cell and COVID-19 [12]. ACS in a pediatric patient does not necessarily portend a poor outcome [13, 14]. Subsequent data have challenged the notion that sickle cell disease is protective [15, 16]. Genetic data suggests that sickle cell disease may be a risk factor for COVID-19 pneumonia [17]. Exchange transfusion has been reported in these patients with good clinical outcomes, although the numbers are limited [10, 18]. Good outcomes have also been reported in case reports of both adult and pediatric patients with severe ACS in the setting of COVID-19 receiving tocilizumab, an IL-6 inhibitor [19, 20]. These data were not available at the time of treatment for our patient nor was the clinical entity of Multisystem Inflammatory Syndrome in Children (MIS-C) known [21]. Bilateral pulmonary emboli have been reported in pediatric COVID-19 patients with sickle cell but none associated with death [20].

Alpha-thalassemia trait appears to have a protective effect in the setting of HbSS disease with regard to red blood cell metrics [22]. Despite this, the clinical effects of sickle cell disease and alpha-thalassemia trait (or alpha-thalassemia disease) are not clear [22]. The effects of alpha-thalassemia on susceptibility to severe COVID-19 infection are unknown [23]. In addition, HbSC disease is milder than HbSS disease including lower rates of ACS [24, 25] although HbSC patients may be at particular risk for fat embolism syndrome [26].

This patient died of acute hypoxemic respiratory failure, but without an autopsy, it is difficult to determine the exact mechanism. Let us hypothesize that our patient died from refractory hypoxemia secondary to severe V/Q mismatch in the setting of severe COVID-19 inflammatory response leading to purulent, thick secretions of the alveoli and airway. Copious secretions may have occluded the endotracheal tube or prevented adequate gas exchange at the alveolar level despite increased ventilator settings. Pulmonary emboli are less likely given the patient was receiving prophylactic anticoagulation. Fat emboli are a possible source given his underling hematological diagnosis but would require autopsy for confirmation. Tension pneumothorax is unlikely given the lack of tachycardia, hypotension, or evacuation of air with needle chest decompression. Early in the pandemic, our institution was not offering high-frequency oscillatory ventilation or extracorporeal membrane oxygenation in SARS-CoV-2-positive pediatric patients although it is unclear if either procedure would have altered the course for this child.

A dedicated approach to the management of children at risk for severe ACSin the setting of COVID-19 is necessary. Guideline-based care includes remdesivir for patients not mechanically ventilated and a short course of steroids for severe or critical COVID-19 [27]. We would maximize airway clearance including early noninvasive positive pressure, incentive spirometry, open suctioning when intubated (versus only inline suctioning as we did), early mobilization (physical and occupational therapy), and chest percussion therapy. Patients requiring noninvasive positive pressure should receive remdesivir while mechanically ventilated patients should follow a high PEEP strategy [27]. Special attention in sickle cell disease patients with COVID-19 should include venous thromboembolism prophylaxis, conservative fluid management, and avoidance of hypoxemia to decrease the chances of worsening ACS [27]. Notably, this child was discharged from the ED after his initial presentation. The patient and his family remained in close contact with the hematology team given his elevated risk for ACS and rapid decompensation. This allowed a rapid referral back to the ED in the setting of worsening symptoms which is consistent with current recommendations [28]. Dedicated patient pathways and clinical protocols are vital for children with hemoglobinopathies [23].

Although children have significantly better outcomes than adults with COVID-19, pediatric intensivists must remain vigilant. This report of a pediatric death in a patient with HbSC and alpha-thalassemia trait with severe COVID-19 following an exchange transfusion underscores the risk of pediatric death when treating any patient with a hemoglobinopathy during the SARS-CoV-2 pandemic [2, 29, 30].

## Figures and Tables

**Table 1:** Notable lab values. Peak (or nadir) noted as clinically relevant and values on day of arrest.

Lab	Peak (nadir)	Day of arrest	Reference (units)
Procalcitonin (on presentation, not repeated)	0.31	—	≤0.08 (ng/mL)
C-reactive protein	127	—	0.00-10.00 (mg/L)
Ferritin (on presentation, not repeated)	250	—	30.0-400.0 (ng/mL)
D-dimer	>20.00	18.21	≤0.80 (*μ*g/mL FEU)
Fibrinogen	458	458	191–430 (mg/dL)
International normalization ratio	1.7	1.3	0.9–1.1
Partial thromboplastin time	52.8	44.8	23.9–34.7 (seconds)
White blood cell count	26.9	23.54	3.84–9.84 (×10^−3^/*μ*L)
Band	24%	0%	0–0 (%)
Lymphocytes	6% (nadir)	11.1%	16.4-52.7 (%)
Neutrophils	77%	76.4%	32.5–74.5 (%)
Monocytes	3% (nadir)	11.1%	4.4–12.3 (%)
Eosinophils	0.1%	0%	0.0–4.0 (%)
Basophils	1%	0.1	0.0–0.7 (%)
Hemoglobin	8 (nadir)	8	(g/dL)
Platelets	36 (nadir)	126	175–332 (×10^3^/*μ*L)
Partial thromboplastin time	52.8	44.8	23.9–34.7 seconds
Creatinine (baseline 0.66)	1.6	1.33	0.60-1.00 mg/dL
Interleukin-18	757	—	89-540 pg/mL
CXCL9	136	—	≤121 pg/mL
Interleukin-6	78.7	—	≤5 pg/mL
Interleukin-10	59	—	≤18 pg/mL
Creatine kinase	950	—	64.0-499.0 U/L
Troponin-T	6	—	≤22 ng/L

## Data Availability

The essential data are provided in the manuscript. There is no additional data or material.

## References

[1] Bailey L. C., Razzaghi H., Burrows E. K. (2020). Assessment of 135 794 pediatric patients tested for severe acute respiratory syndrome coronavirus 2 across the United States. *JAMA pediatrics*.

[2] Shekerdemian L. S., Mahmood N. R., Wolfe K. K. (2020). Characteristics and outcomes of children with coronavirus disease 2019 (COVID-19) infection admitted to US and Canadian pediatric intensive care units. *JAMA Pediatrics*.

[3] Yawn B. P., Buchanan G. R., Afenyi-Annan A. N. (2014). Management of sickle cell disease: summary of the 2014 evidence-based report by expert panel members. *Journal of the American Medical Association*.

[4] Vichinsky E. P., Neumayr L. D., Earles A. N. (2000). Causes and outcomes of the acute chest syndrome in sickle cell Disease. *The New England Journal of Medicine*.

[5] The Pediatric Acute Lung Injury Consensus Conference Group (2015). Pediatric acute respiratory distress syndrome: consensus recommendations from the Pediatric Acute Lung Injury Consensus Conference. *Pediatric Critical Care Medicine*.

[6] Ferguson N. D., Fan E., Camporota L. (2012). The Berlin definition of ARDS: an expanded rationale, justification, and supplementary material. *Intensive Care Medicine*.

[7] Piazza G., Morrow D. A. (2020). Diagnosis, management, and pathophysiology of arterial and venous thrombosis in COVID-19. *Journal of the American Medical Association*.

[8] Chakravorty S., Padmore-Payne G., Ike F. (2020). COVID-19 in patients with sickle cell disease - a case series from a UK Tertiary Hospital. *Haematologica*.

[9] McCloskey K. A., Meenan J., Hall R., Tsitsikas D. A. (2020). COVID-19 infection and sickle cell disease: a UK centre experience. *British Journal of Haematology*.

[10] Hussain F. A., Njoku F. U., Saraf S. L., Molokie R. E., Gordeuk V. R., Han J. (2020). COVID-19 infection in patients with sickle cell disease. *British Journal of Haematology*.

[11] Sahu K. K., George L., Jones N., Mangla A. (2021). COVID-19 in patients with sickle cell disease: a single center experience from Ohio, United States. *Journal of Medical Virology*.

[12] Arlet J. B., de Luna G., Khimoud D. (2020). Prognosis of patients with sickle cell disease and COVID-19: a French experience. *The Lancet Haematology*.

[13] Morrone K. A., Strumph K., Liszewski M. J. (2020). Acute chest syndrome in the setting of SARS-COV-2 infections-a case series at an urban medical center in the Bronx. *Pediatric Blood & Cancer*.

[14] Elia G. M., Angel A., Regacini R. (2021). Acute chest syndrome and COVID-19 in sickle cell disease pediatric patients. *Hematology, Transfusion and Cell Therapy*.

[15] Minniti C. P., Zaidi A. U., Nouraie M. (2021). Clinical predictors of poor outcomes in patients with sickle cell disease and COVID-19 infection. *Blood Advances*.

[16] Vilela T. S., Braga J. A. P., Loggetto S. R. (2021). Hemoglobinopathy and pediatrics in the time of COVID-19. *Hematology, transfusion and cell therapy*.

[17] Chen H. H., Shaw D. M., Petty L. E. (2021). Host genetic effects in pneumonia. *American Journal of Human Genetics*.

[18] Beerkens F., John M., Puliafito B., Corbett V., Edwards C., Tremblay D. (2020). COVID-19 pneumonia as a cause of acute chest syndrome in an adult sickle cell patient. *American Journal of Hematology*.

[19] de Luna G., Habibi A., Deux J. F. (2020). Rapid and severe Covid-19 pneumonia with severe acute chest syndrome in a sickle cell patient successfully treated with tocilizumab. *American Journal of Hematology*.

[20] Odièvre M. H., de Marcellus C., Ducou le Pointe H. (2020). Dramatic improvement after tocilizumab of severe COVID-19 in a child with sickle cell disease and acute chest syndrome. *American Journal of Hematology*.

[21] Cheung E. W., Zachariah P., Gorelik M. (2020). Multisystem inflammatory syndrome related to COVID-19 in previously healthy children and adolescents in New York City. *Journal of the American Medical Association*.

[22] Higgs D. R., Aldridge B. E., Lamb J. (1982). The interaction of alpha-thalassemia and homozygous sickle-cell disease. *The New England Journal of Medicine*.

[23] Farmakis D., Giakoumis A., Cannon L., Angastiniotis M., Eleftheriou A. (2020). COVID-19 and thalassaemia: a position statement of the Thalassaemia International Federation. *European Journal of Haematology*.

[24] Ballas S. K., Lewis C. N., Noone A. M., Krasnow S. H., Kamarulzaman E., Burka E. R. (1982). Clinical, hematological, and biochemical features of Hb SC disease. *American Journal of Hematology*.

[25] Castro O., Brambilla D. J., Thorington B. (1994). The acute chest syndrome in sickle cell disease: incidence and risk factors. The Cooperative Study of Sickle Cell Disease. *Blood*.

[26] Scheifer C., Lionnet F., Bachmeyer C. (2017). Cerebral fat embolism in hemoglobin SC disease. *The American Journal of Medicine*.

[27] Alhazzani W., Evans L., Alshamsi F. (2021). Surviving sepsis campaign guidelines on the management of adults with coronavirus disease 2019 (COVID-19) in the ICU: first update. *Critical Care Medicine*.

[28] (2020). America SCDAo. Sickle cell disease and COVID-19: provider advisory. https://www.sicklecelldisease.org/2020/03/18/sickle-cell-disease-and-covid-19-provider-directory/.

[29] Hoang A., Chorath K., Moreira A. (2020). COVID-19 in 7780 pediatric patients: a systematic review. *EClinicalMedicine*.

[30] Lu X., Zhang L., du H. (2020). SARS-CoV-2 infection in children. *The New England Journal of Medicine*.

